# Mycelium-Based Composite Graded Materials:  Assessing the Effects of Time and Substrate Mixture on Mechanical Properties

**DOI:** 10.3390/biomimetics7020048

**Published:** 2022-04-19

**Authors:** Ali Ghazvinian, Benay Gürsoy

**Affiliations:** Department of Architecture, Penn State University, University Park, PA 16802, USA; bug61@psu.edu

**Keywords:** mycelium-based composites, compressive structures, compressive strength, digital image correlation, masonry

## Abstract

Mycelium-based composites (MBC) are biodegradable, lightweight, and regenerative materials. Mycelium is the vegetative root of fungi through which they decompose organic matter. The proper treatment of the decomposition process results in MBC. MBC have been used in different industries to substitute common materials to address several challenges such as limited resources and large landfill waste after the lifecycle. One of the industries which started using this material is the architecture, engineering, and construction (AEC) industry. Therefore, scholars have made several efforts to introduce this material to the building industry. The cultivation process of MBC includes multiple parameters that affect the material properties of the outcome. In this paper, as a part of a larger research on defining a framework to use MBC as a structural material in the building industry, we defined different grades of MBC to address various functions. Furthermore, we tested the role of substrate mixture and the cultivation time on the mechanical behavior of the material. Our tests show a direct relationship between the density of the substrate and the mechanical strength. At the same time, there is a reverse relation between the cultivation time and the material mechanical performance.

## 1. Introduction

The architecture, engineering, and construction (AEC) industry consumes half of the mineral resources and contributes the most to landfill waste [1]. Therefore, there is a need for alternative construction materials and greener energy resources to reduce the AEC industry’s global greenhouse gas emissions and landfill waste. A circular approach must replace the current linear approach of extract-produce-use-dump. This approach emphasizes reuse, remanufacturing, refurbishment, repair, and upgrading of materials and utilization of solar, wind, biomass, and waste-derived energy during the product’s life cycle. [2]. One possible path to conform with circular economies is through the use of bio-based materials as alternatives to conventional materials in construction [3,4]. These materials can be produced using waste as one of their initial ingredients and can become reusable, recyclable, or compostable at the end of their lifecycles.

Due to the rapid population growth worldwide, the global demand for food and agricultural wastes and byproducts has increased [5]. Besides, this growing population needs affordable habitat. Since the traditional ways of dumping agricultural waste into landfills or burning them impact global warming [6], converting agricultural waste into building components seems an optimal solution for these problems. Various bio-based materials are studied in this context [7]. The development of these materials can be costly, time-consuming, and inefficient due to the problematic methods of processing and functionalization [8], although they have a multitude of advantages. One of these materials is mycelium-based composites (MBC). MBC is manufactured using a low-energy and natural process that sequesters carbon and uses waste as the input. There are several applications of mycelium-based matter and fungal biotechnologies [9]. This research focuses on using MBC as load-bearing masonry components in construction.

## 2. Background

Mycelium is the vegetative root of fungi. Mycelium has a long, branching, and filamentous structure called hyphae that secrete enzymes to break down the biopolymers into simpler bodies of digestible carbon-based nutrients. The outcome of this process is an organic colony of hyphae. The organic matter bounds with this hyphal structure and forms fungal skin during this process. When this process is ceased through drying or heating, the incomplete process results in MBC. This composite material is made of the substrate as the filler and the hyphal mycelium as the binder. Without the hyphal binder, the substrate works as an inconsistent mass of particles and shows negligible mechanical performance. This bio-based composite can be shaped to produce panels, bricks, or various objects [10]. The properties of MBC depend on various cultivation parameters, such as the fungal species, substrates, growth conditions, processing of material, and additives [5,11]. This dependence on the controllable parameters enables MBC to meet specific application requirements [5]. Among these applications are acoustic insulation [12,13], thermal insulation [14,15,16,17], packaging [18,19,20,21], fire retardants [22,23,24], and structural building components [11,25,26,27,28,29,30,31,32,33,34,35]. 

Scholars are making various efforts to make MBC meet the performance requirements of the AEC industry. One approach is to enhance the material properties of MBC by investigating the cultivation parameters. Another approach is to develop novel design and fabrication techniques around the specific material properties of MBC via geometry and form optimizations [32]. This paper focuses on the former approach. 

### 2.1. The Cultivation Process of MBC

**Figure 1:**
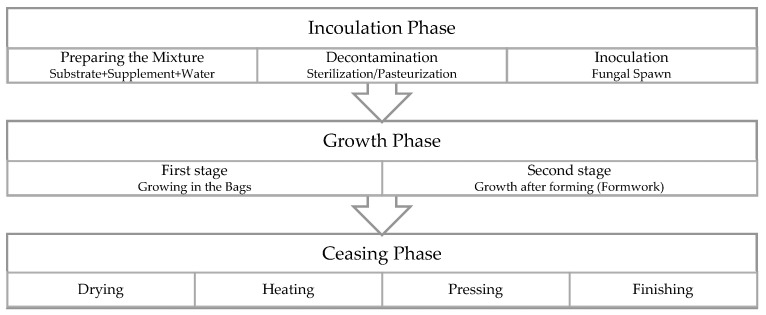
MBC cultivation process.

The cultivation process of MBC has three major phases: inoculation, growth, and ceasing. Figure 1 illustrates the cultivation process of MBC. 

The substrate mixture is first prepared with organic matter as the filler, optional supplements that provide additional carbon and nitrogen, and water in the inoculation phase. This mixture is then pasteurized or sterilized to eliminate any other living organism that can pose a threat to mycelium growth. The substrate mixture is typically placed within autoclavable bags and heated in autoclave machines for less than an hour at 121 °C. Alternative methods such as the use of herbal remedies [15] and heating in lower temperatures for longer durations are also available. Once the sterilization is complete, the substrate mixture is cooled down to room temperature, and the fungal spawns are added. The ingredients of the substrate mixture and the fungal species used in inoculation affect the material properties of MBC [36,37]. The second phase of the MBC cultivation process, the growth, starts in the autoclavable bags. Depending on the application, growth can continue within formworks [38,39]. Some studies explored the extrusion of the substrate mixtures through additive manufacturing [40,41,42,43,44,45,46,47,48]. Some of the parameters that affect the resulting material properties in this phase are the duration of growth, environmental conditions such as temperature and relative humidity, and CO_2_ concentration [49,50]. The third phase, ceasing, also influences the resulting MBC. In this phase, mycelial growth is stopped by either drying or heating the colonized substrate mixture. Researchers have also explored experimental processes such as rubbing herbal oils and hot or cold pressing the composite. The method of ceasing and parameters associated with the chosen method (for example, pressing temperature) [51] can alter the material properties of MBC.

### 2.2. Role of Cultivation Parameters on the Mechanical Behavior of MBC

Various scholars have studied the mechanical behavior of MBC, specifically compressive strength. The consensus is that the mechanical behavior of MBC is comparable to that of synthetic foams, with room for enhancement [10]. MBC made of low-weight substrate mixtures have similar compressive strength to polystyrene foams and are weaker than polyurethane and phenolic formaldehyde resins [5].

Among the various research that explore the role of cultivation parameters on the outcome, Holt et al. (2012) [20] studied the substrate mixtures of six different cotton plant biomass. Yang et al. (2017) [7] experimented with the degree of compaction of substrates within formworks. They also tested the role of the duration of cultivation (two and six weeks) on the outcome. Their results show that the densely packed samples have higher compressive strength and elastic moduli. In comparison, the longer duration of cultivation results in better compressive strength and lower elastic moduli. Islam et al. (2018) [52] defined three sizes: small (from 0.4 to 0.9 mm), medium (from 0.9 to 1.7 mm), and large (from 1.7 to 6.7 mm) fillers (such as sawdust), and a mixture of these three to study the effects of filler size on compressive strength. They reported that the mechanical behavior of MBC is not affected by the filler size. On the contrary, the experiments by Elsacker et al. (2019) [53] show that fiber size is more influential on the mechanical strength than the type of fibrous substrate used. Except for the dust material that yielded poor growth and mechanical properties, the more chopped material resulted in better strength. In conformation with Yang et al. (2017) [7], their experiments showed that densely packed substrates had better mechanical properties than loose fibers. 

Attias et al. (2019) [54] experimented with three different spawns and two growing protocols. They used *Colorius*, *Trametes*, and *Ganoderma* species and cultivated them with a 7-day difference in incubation time to establish their final experiments on the suitability of these conditions. They continued their study in Attias et al. (2020) [25] and reported better mechanical behavior for the samples cultivated with *Ganoderma* species. They also reported a reverse relation between the mycelium colonization and compressive strength, suggesting that shorter incubation periods restrict the organic matter digestion and preserve the mechanical characteristics of the substrate mixture. On the other hand, longer incubation times change the material content of the digestible substrate more and weaken the produced MBC. Bruscato et al. (2019) [55] utilized three different species of *Pycnoporus sanguineus*, *Pleurotus albidus*, and *Lentinus velutinus* for cultivation with sawdust and wheat bran. They found *L. velutinus* to be resulting in weaker composites because of the way mycelium colonizes during the growth. They suggest that the colonization of this species is more accentuated around the interstices of the mixture fillers than the overall agglomerate, which is different from the other two species, and that this is the reason for lower mechanical strength. 

Appels et al. (2019) [50] Studied the role of different species, substrates, and pressing conditions on material behavior. They tested the bending capacity of MBC in their studies, with *T. multicolor* and *P. ostreatus* growing on rapeseed straw and beech sawdust. They also used three different conditions for ceasing: non-pressed, cold-pressed, and heat-pressed. Their most important result is the direct relation of mechanical strength and elastic moduli with the pressing, mainly through hot-pressing. They reported that heat-pressing shifts MBC performance from foam-like to wood-like. They also explained that colonization of mycelium occurs better at the outer parts of the substrates than the cores, emphasizing the importance of forcing air through the center of the substrate. Additional research on the optimal temperatures for hot-pressing the substrates reveals that lower temperatures result in weaker materials, and higher temperatures may burn the materials [51]. 

Ghazvinian et al. (2019) [28] studied the role of supplements on two different substrates with *P. ostreatus*. Their results show slightly stronger materials with 7% wheat bran in the inoculation phase. There is also a considerable difference between MBC cultivated with oak sawdust and wheat straw. Ongpeng et al. (2020) [33] utilized clay, rice bran, and sawdust mixed with different waste materials to make MBC bricks and tested them to compare with masonry minimum limits. They also used the compressed substrates without mycelium to study the role of mycelium as the binding agent in these bricks. For the clay samples, the mycelium content was not modifying the characteristics, while for the other samples, mycelium bound the substrates, which resulted in stronger materials. Besides, all the mycelium-based bricks passed the minimum compressive strength for masonry bricks. One other important aspect studied by Zimele et al. (2020) [27] is the biodegradability of this material after use. Compared to hemp magnesium oxychloride concrete and cemented wood wool panel, two other bio-based materials, MBC showed quadruple biodegradability. This biodegradability is a testimony of the circularity of MBC when used in the AEC industry. An LCA analysis of MBC bricks on the lab and industrial scale shows reductions in most impact categories. Biodegradability might reduce the AEC industry’s environmental footprint if conventional building materials can be substituted with MBC [56].

In a more recent study, Elsacker et al. (2021) [57] investigated the addition of other organisms, such as bacterial cellulose to *T. versicolor* inoculated on hemp-based substrates to make particleboards. They found the enhancing role of bacterial cellulose in improving internal bonding. 

## 3. Materials and Methods

This paper studied the effects of three different MBC cultivation parameters on compressive strength. The first parameter studied is the substrate mixture type. We created various mixtures by combining particle-based (i.e., sawdust) and fibrous (i.e., straw) materials. The other two parameters we studied are related to the duration of cultivation. The entire and partial cultivation time in bags and formworks has been investigated.

**Figure 2:**
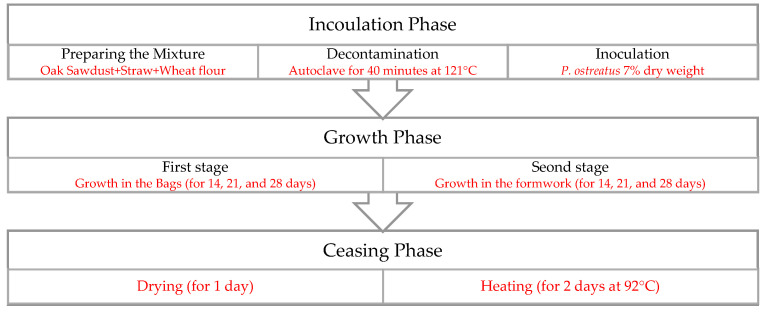
Details of MBC cultivation.

As mentioned before, there are three primary phases in the cultivation process of MBC. Figure 2 illustrates the materials and methods employed in these phases as part of this research.

### 3.1. Substrate Mixtures Preparation

Five different substrate mixtures are created for the experiment based on the sawdust to straw ratios. Oakwood pellets (Atlanta, GA, USA) and wheat straws (chopped, 3 cm long) are the base materials used for the mixtures. The various mixtures have straw and sawdust ratios of 1 to 1, 2, 3, 7, and one with sawdust only, as shown in Table 1. In addition, unbleached whole wheat flour (Bentonville, AR, USA) has been added to the mixtures by 7% of the dry weight to enhance the growth process. The water content of the mixtures has been controlled to stay between 65% to 70%, following the best practices for *Pleurotus ostreatus* mushroom cultivation.

The five substrate mixtures have been hand-mixed for 120 s to distribute the ingredients and water evenly. They were then moved to autoclavable bags (Impresa Mushroom Growing Bags) for sterilization and test paper bags for humidity check. Bags were sterilized in the autoclave machine at 121 °C temperature for 40 min and then removed from the autoclave machine and left to cool down (overnight, at room temperature).

### 3.2. Cultivation of Materials

The fungal spawns of *Pleurotus ostreatus* were purchased from local suppliers (Lambert Spawn, Coatesville, PA, USA). Oyster mushroom spawn has been used because 1) it is widely available locally, and 2) satisfactory results have been obtained with similar genera according to the literature [8]. Sterilized substrates were inoculated with the spawns by 7% of the dry weight in a sterilized environment. The bags were then placed in a growth room with environmental control. The temperature was set to 21 °C, and the relative humidity to 95%. The room was kept dark to help with the growth process.

To study the effects of different durations of growth on the mechanical behavior of MBC, three different durations of growth for 5, 6, and 7 weeks were selected, regarding the best results from the literature [7]. The growth process of MBC has been divided into two phases: within bags and formworks. To compare the partial growth of MBC in bags and formworks, the substrate mixtures have been placed in formworks at different times. These different substrate mixtures and timeframes made 35 different treatments. The treatments are coded as X_ij:_ X indicates the substrate mixture used for the cultivation, and i and j indicate the cultivation time (weeks) in bags and formworks. The details about the total and partial duration of the treatments are shown in Table A1.

### 3.3. Preparing Samples for Mechanical Test

The formworks for this experiment were made of cardboard covered with plastic tapes to avoid the hyphae feeding on the cardboard and decrease the cardboard’s humidity level by capillary action. All formworks were cubes of 5 cm in length. The materials have been moved to the formworks after the first growth phase in the bags. We filled the formworks in three steps and hand-pressed the materials at each step. To control the conditions of similar samples for later experiments, the amount of material added in each step to the formwork was controlled by weight. For example, while all the formworks that were filled for samples A_ij_ weighed 120 g in total, each formwork was filled in three stages, adding 40 g of the material at each stage. Finally, the formworks were placed in the same room for the second phase of the growth process.

Following the growth phase, the samples were unmolded and placed in the oven for 48 h at 92 °C. After this heating process, almost all samples lost about two-thirds of their weight, showing that they were thoroughly dried and ceased the growth process. The cubic samples were then moved to the lab for the mechanical tests.

### 3.4. Mechanical Tests

**Figure 3:**
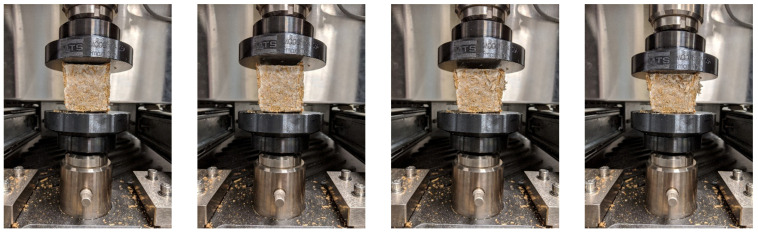
Mechanical tests were performed on MBC sample C_33_ with a 0.05 mm per second rate with the MTS machine (Figures are taken at 15-s intervals).

The mechanical tests were performed on an MTS machine (Figure 3). ASTM C109 standard procedure requires testing three samples of each treatment. Therefore, for each treatment, we have created three samples. Each sample has been compressed to 80% of its initial height with a 0.05 mm per second rate to study its behavior under compression. We considered material strength as the stress in which material collapsed (the peak stress in stress-strain diagrams when a peak occurs) or the stress at the 10% strain, whichever comes first. This paper reports the average result of each sample group when the difference is less than 8.7%.

**Figure 4:**
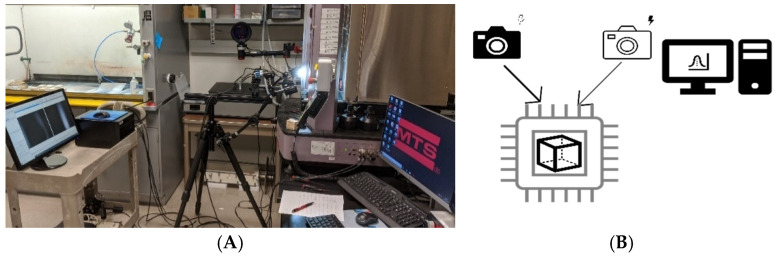
(**A**) The Digital Image Correlation (DIC) system and (**B**) its scheme. DIC includes two cameras that capture the alterations of the sample while under compression and correlate the images.

For the treatments X_24_, X_33_, and X_42_, the Correlated Solutions VIC-3D Digital Image Correlation (DIC) system was also used to enable a more detailed study of MBC behavior under compression (Figure 4). In this system, a setup with two (or more) cameras captures images from the samples while they are reacting to an external force or stimulus. The samples are prepared with speckle points, lights, or other readable signs. After the experiment is conducted, the images from the cameras are correlated to present the alterations of the samples throughout the process. The DIC shows the exact deformations of the sample and enables studying the quantitative and qualitative mechanical behavior.

## 4. Results and Discussion

### 4.1. The Effects of Sawdust to Straw Ratio in Substrate Mixtures on Compressive Strength

Five substrate mixtures (A, B, C, D, and E) with different ratios of sawdust and straw have been prepared (Table 1). Each mixture has been subjected to seven different differential growth times, resulting in 35 treatments. Table 2 shows the results of the mechanical tests for all 35 treatments. The treatments with only sawdust content (A) showed the best mechanical behavior. This result is in line with the literature [50,57]. While the treatments with 1 to 1 sawdust to the straw ratio (E) showed the weakest mechanical behavior and lowest compressive strength, the other three substrate mixtures with sawdust to straw ratios of 1 to 2 (D), 1 to 3 (C), and 1 to 7 (B) exhibited negligible differences. 

The test results show that stronger substrates with more lignin content, such as sawdust, result in MBC with better compressive strengths, while weaker substrates, such as straw, result in MBC with weaker compressive strengths. There is a direct correlation between the density of the substrate, the density of MBC, and the mechanical strength of the material. However, the difference in the mechanical strength of MBC cultivated with substrates that include both straw and sawdust is negligible.

### 4.2. The Effects of Total Growth Time on Compressive Strength

**Figure 5:**
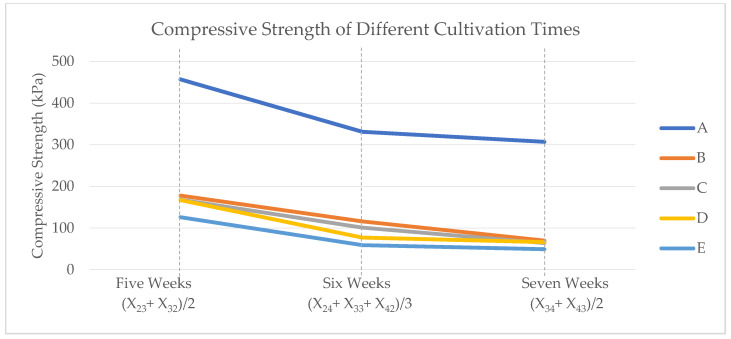
Compressive strength (kPa) of treatments with different growth times.

Substrate mixtures have been grown for five (X_23_, X_32_), six (X_24_, X_33_, X_42_), and seven (X_34_, X_43_) weeks. According to the literature [25], the longer growth time causes more organic substrate degradation, which means less substrate and more hyphal structures. Since most of the compressive strength of MBC is from the substrates, longer growth times result in less compressive strength. The results from our mechanical tests are also in line with the literature [7,25]. Figure 5 shows the average compressive strength for each substrate mixture (A, B, C, D, and E) grown for five, six, and seven weeks. For each substrate mixture, the compressive strength decreases by increasing the total cultivation time.

### 4.3. The Effects of Varied Bag/Formwork Growth Times on Compressive Strength

**Figure 6:**
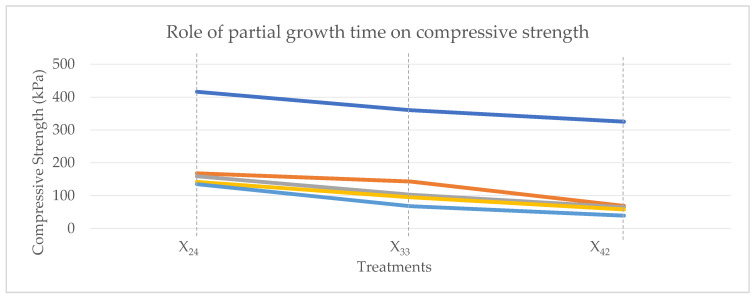
Compressive strength (kPa) of treatments with the six-week cultivation time and different partial growth times.

Treatments with the exact total growth times have been grown for different time frames within bags and formworks. We tested this feature to find the optimal duration of growth in each phase of MBC cultivation. For each substrate mixture (A, B, C, D, and E), we have two sets of treatments cultivated for five weeks (X_23_ and X_32_), three sets for six weeks (X_24_, X_33_, and X_42_), and two for seven weeks (X_34_ and X_43_). Our tests show that more extended cultivation in formworks yields better mechanical performance than longer cultivation times within bags for all substrate mixtures and cultivation sets. Figure 6 shows the average compressive strength of all the substrate mixtures cultivated for six weeks (X_24_, X_33_, and X_42_) regarding the cultivation time. The samples grown in molds for a more extended time show better mechanical performance. 

The duration of cultivation has an inverse relation to the compressive strength of the material. We cultivated mixtures for 5, 6, and 7 weeks. The material cultivated for five weeks showed higher compressive strength in all the cases. This result is in accordance with the published literature [7]. For all the samples, the extended time of growth in formworks compared to the growth in bags yielded stronger compressive strengths.

### 4.4. Digital Image Correlation (DIC)

For the cubic samples of treatments X_24_, X_33_, and X_42_, we used the DIC setup with two 5 MP (2448 × 2048 pixels by 50 fps) and two Schneider Xenoplan 1.9/35 mm compact series lenses. This setup lets us capture the behavior of cubic samples under compression. The cubic samples were speckled prior to the test, and the speckles’ movement during the test was monitored. This monitoring allowed us to have a more detailed quantitative study of the material’s mechanical behavior and qualitatively study its behavior. The detailed movement data of speckles enables access to different displacement and strain amounts happening throughout the loading process. Mapping these on the loading timeline enables us to have more precise results. First, the samples’ mechanical strength and elastic moduli were calculated using the DIC system data (reported in Table A1). These results verified the results from the extensometer attached to the MTS machine with less than a 5% difference. The system also allowed us to calculate other engineering characteristics of the material, such as the principal strains, shear moduli, and the Poison ratio. Besides that, the images taken from the system and the correlation between images reveal how the material behaves under compression.

**Figure 7:**
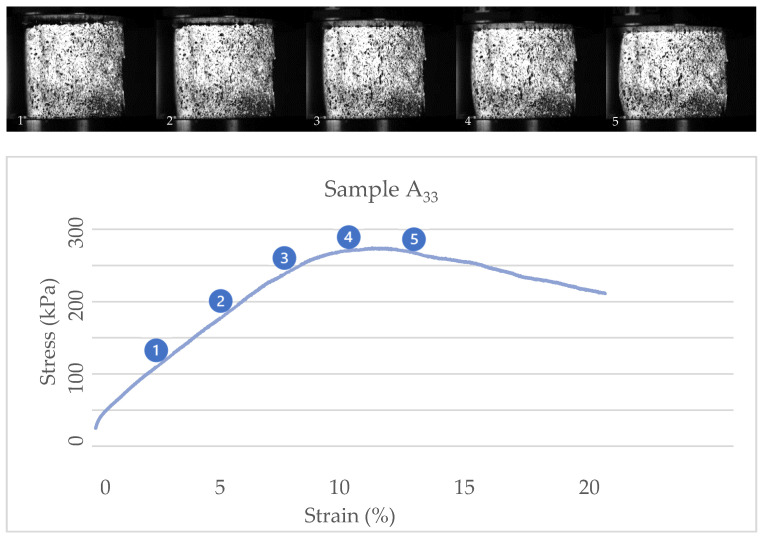
Stress-strain diagram and actual images of cubic sample A_33_.

**Figure 8:**
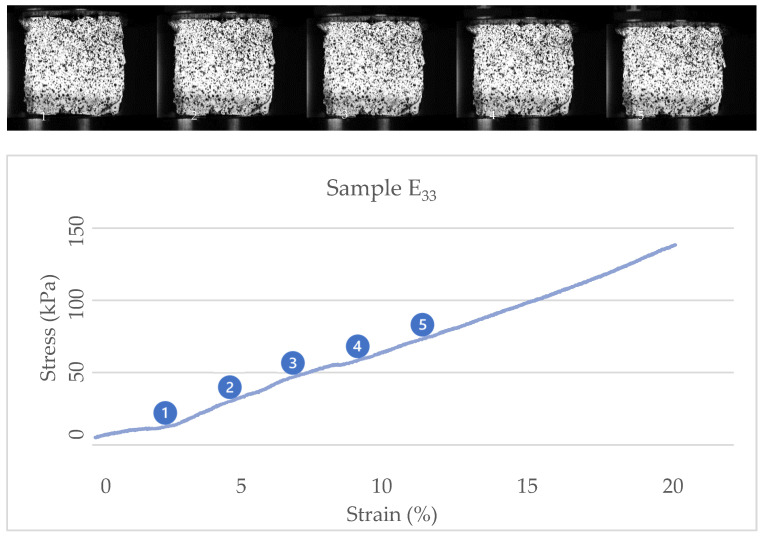
Stress-strain diagram and actual images of cubic sample E_33_.

One of the results from the tests and the study of the images show that treatments with substrate mixtures with more sawdust content (Sample A) behave with toughness and show a peak in their stress/strain diagrams. In comparison, treatments with more straw content in their substrate mixtures (Sample E) behave with hardness and reach the fracture point without showing plastic behaviors. Figure 7 and Figure 8 show the stress/strain diagrams of cubic samples of treatments A_33_ and E_33 and_ some images of their behavior under compression.

The images show that the cubic sample of the A_33_ treatment develops a crack in the center and deforms before reaching the peak point (near its 10% strain). In comparison, the sample of the E_33_ treatment does not show large cracks and reaches the maximum strain without fracture. Most treatments with substrate mixtures with more straw content have shown this behavior. 

**Figure 9:**
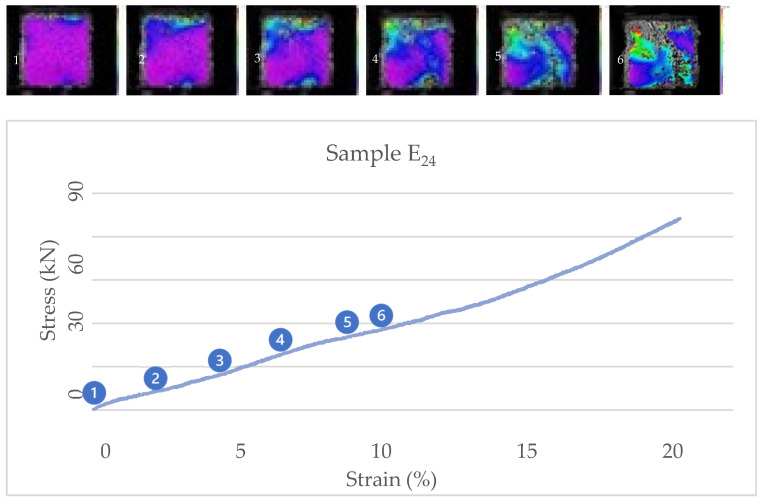
Stress-strain diagram and correlated images of cubic sample E_24_.

**Figure 10:**
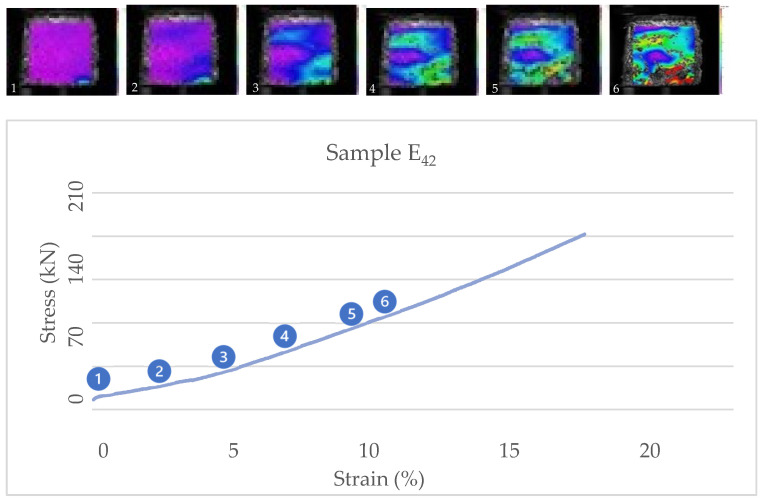
Stress-strain diagram and correlated images of sample E_42_.

Figure 9 and Figure 10 show the correlation of images from cubic samples E24 and E42 and their stress/strain diagrams. The other samples of substrate mixture E show the same behavior. Studying the correlated images and the internal strains of the cubic samples also show that the material works in compression with more tendency to use its toughness.

The results from the DIC system showed that substrate mixtures with more sawdust content tend to use their hardness, while straw-based substrate mixtures tend to behave with toughness. As tough materials are more resistant to fracturing and are not easily breakable, they seem to be better options for the compressive structural systems working through form. While, for functions that need materials that bear the load by their strength, materials with hardness tendencies are preferable.

## 5. Conclusions 

The sustainable aspects of MBC make them suitable alternatives for their non-sustainable counterparts in several industries. From foam-like materials in the packaging industry to panels in the building industry, MBC cover many functions with different performance parameters. As studied in this paper, the AEC industry can also benefit more from the mechanical strength of MBC. These composites offer lightweight, graded, and biodegradable alternatives to conventional building materials and can help address the environmental problems caused by the AEC industry. 

This research used agricultural waste (sawdust and straw) to cultivate MBC using locally available fungal species. We presented experiments in which we prepared treatments with five different substrate mixtures of varying sawdust to straw ratios. We tested the effects of the total duration of growth on the compressive strength of MBC cultivated with these treatments. We also tested the effects of varying the duration of growth in bags and the duration of growth in formworks on compressive strength. Our mechanical tests showed the possibility of cultivating a gradient of compressive materials. The results are also verified with a Digital Image Correlation (DIC) system, which also enabled the extraction of other material characteristics for future use in structural form-finding and the study of the qualitative behavior of the samples under compression. 

MBC material is lightweight, its dead load is negligible, and it bears the load through the form. So, material grades with tougher properties can enable the designing and building of compressive structural forms. Besides, the harder grades can be used for the functions that bear the light loads through the strength of materials. In the following stages of this research, our goal is to develop computational form-finding methods to design and fabricate compressive structures with MBC that employ the results of our mechanical tests as the main inputs in optimizing the structural forms. 

## Figures and Tables

**Table 1:** Characteristics of different mixtures.

Mixture	Sawdust Ratio	Straw Ratio	Water Content	Wheat Flour Content	*Fungal* sp. Content
A	1	0	65–70%	7% DW	7% DW
B	1	7	65–70%	7% DW	7% DW
C	1	3	65–70%	7% DW	7% DW
D	1	2	65–70%	7% DW	7% DW
E	1	1	65–70%	7% DW	7% DW

DW = dry weight of the mixture.

**Table 2:** Compressive strength of different treatments (kPa).

	Treatments							
**Substrate** **Mixtures**		X_23_	X_24_	X_32_	X_33_	X_34_	X_42_	X_43_
	A	498	416	330	360	303	325	288
	B	187	168	107	143	97	69	71
	C	177	159	118	103	82	65	62
	D	192	142	62	95	74	58	73
	E	116	135	34	68	75	39	58

## Data Availability

Data might be requested by contacting the corresponding author.

## Appendix A

The table below shows the characteristics of treatments used for the experiments and their mechanical strength and elastic moduli regarding the DIC results.

**Table A1:** Characteristics of Treatments and their Mechanical Properties.

Treatment	Substrate Mixture	Growth in Bags (Days)	Growth in Formwork (Days)	Total Growth Time (Days)	Mechanical Strength (kPa)	Elastic Moduli (MPa)
A_23_	A: Sawdust only 65–70% Water Content 7% DW Wheat Flour 7% DW *Fungal* sp.	14	21	35	491	6.1
A_24_		14	28	42	418	3.4
A_32_		21	14	35	335	3.3
A_33_		21	21	42	355	3.5
A_34_		21	28	49	300	3.4
A_42_		28	14	42	321	3.4
A_43_		28	21	49	292	3.0
B_23_	B: Straw to Sawdust = 7/1 65–70% Water Content 7% DW Wheat Flour 7% DW *Fungal* sp.	14	21	35	190	1.8
B_24_		14	28	42	170	1.6
B_32_		21	14	35	110	1.1
B_33_		21	21	42	140	0.9
B_34_		21	28	49	98	0.9
B_42_		28	14	42	70	0.8
B_43_		28	21	49	68	0.7
C_23_	C: Straw to Sawdust = 3/1 65–70% Water Content 7% DW Wheat Flour 7% DW *Fungal* sp.	14	21	35	181	1.6
C_24_		14	28	42	161	1.4
C_32_		21	14	35	121	1.7
C_33_		21	21	42	101	0.9
C_34_		21	28	49	81	0.8
C_42_		28	14	42	66	0.8
C_43_		28	21	49	61	0.7
D_23_	D: Straw to Sawdust = 2/1 65–70% Water Content 7% DW Wheat Flour 7% DW *Fungal* sp.	14	21	35	193	0.6
D_24_		14	28	42	142	2.0
D_32_		21	14	35	63	1.5
D_33_		21	21	42	96	0.5
D_34_		21	28	49	75	0.8
D_42_		28	14	42	59	0.7
D_43_		28	21	49	72	0.6
E_23_	E: Straw to Sawdust = 1/1 65–70% Water Content 7% DW Wheat Flour 7% D.W. *Fungal* sp.	14	21	35	120	0.6
E_24_		14	28	42	130	1.3
E_32_		21	14	35	33	1.1
E_33_		21	21	42	69	0.5
E_34_		21	28	49	74	0.7
E_42_		28	14	42	40	0.7
E_43_		28	21	49	60	0.6
